# Early results after surgical treatment of left Ventricular Aneurysm

**DOI:** 10.1186/1749-8090-7-126

**Published:** 2012-11-21

**Authors:** Xisheng Wang, Xuezhi He, Yunqing Mei, Qiang Ji, Jing Feng, Jianzhi Cai, Yifeng Sun, Shiliang Xie

**Affiliations:** 1 The Department of Thoracic Cardiovascular Surgery, Tongji Hospital of Tongji University, 389 Xincun Road, Shanghai, 200065, China; 2 The Department of Thoracic Cardiovascular Surgery, Dalian Municipal Central Hospital, 826 Southwest Road, Dalian, Liaoning, 116033, China

**Keywords:** Left ventricular aneurysm, Liner repair, Endoventricular circular patch plasty, Beating heart

## Abstract

**Background:**

Left ventricular aneurysm (LVA) is a serious complication of myocardial infarction and reduces the chances of survival. Controversy still exists regarding the optimal surgical technique for LVA repair. We analyze the efficacy of two techniques, linear vs. endoventricular circular patch plasty, for repair of LVA and the efficacy of surgical ventricular restoration (SVR) on beating heart.

**Methods:**

This study included 62 patients who underwent SVR from 1086 consecutive patients were subjected to coronary artery bypass grafting (CABG) between 2000 and 2009. All selected patients were divided either into group liner or patch according to the choice of the repair technique depended on factors such as localization, size and dimension of the scar. The patients also were divided either into group beating heart or cardioplegia. The pre-, intra- and postoperative relevant data of all selected patients were analyzed.

**Results:**

The mortality was not significantly different between linear and patch repair groups, also the actuarial survival rates within 24 months (*p*= 0.529). Postoperative echocardiographic findings showed significant improvements in left ventricular function in both groups. The beating heart technique reduced postoperative peak release by 27% for Cardiac troponin I (cTnI) compared with the cardioplegia group (0.46 ± 0.06 ng/mL versus 0.63 ± 0.09 ng/mL, *p*= 0.004), and increased the perioperative survival by 9% (97.2% versus 88.5%), but the actuarial survival rates were not significantly different between the groups from 2 to 24 months (*p*= 0.151).

**Conclusions:**

Both techniques (linear and patch) achieved good results with respect to mortality, functional status and survival. The choice of surgical technique should be adapted in each patient. The beating heart technique may to some extent relieve myocardial injury in patients undergoing SVR.

## Background

Left ventricular aneurysm is a serious complication of myocardial infarction and reduces the chances of survival. Paradoxical motion of the aneurysm reduces left ventricular output and may result in intractable heart failure. A large LVA causes progressive left ventricle (LV) dilatation and volume overload hypertrophy with increased wall tension in the non-infarcted region, decreased LV performance, and thrombus formation in the aneurysmal cavity [1]. Ventricular arrhythmias may develop at the border between healthy and necrotic tissue, due to electrophysiological differences, causing angina or sudden death. When a left ventricular aneurysm leads to pulmonary congestive symptoms, aneurysmectomy may provide relief.

Surgical ventricular restoration has been found to improve cardiac function and functional status in patients with post-infarction LVA, compared to medical therapy alone [2]. Initially, SVR was performed by excision of the aneurysmal area followed by linear repair, this technique left the scarred area of the septum undisturbed [3]. Techniques of geometric repair by endoventricular patch plasty addressed the infarcted septum, resulting in further reduction of LV volume and improved cardiac performance [4].

But controversy still exists regarding the optimal surgical technique for post-infarction LVA repair. We analyze the efficacy of two techniques, linear vs. endoventricular circular patch plasty, for repair of dyskinetic LVA and the efficacy of SVR on beating heart.

## Methods

### Patients

This study included 62 patients who underwent SVR from 1086 consecutive patients were subjected to CABG between 2000 and 2009, by the plication in 1, by the liner repair in 26 (41.9%) and by the endoventricular circular patch plasty repair in 35 (56.5%). Thirty-six (58.1%) were performed on the on-pump beating heart (linear 16, patch 20). All patients had simultaneous coronary revascularization. Clinical characteristics of the patients are shown in Table 1. All patients had previously granted permission for use of their medical records for research purposes, and our institutional committee on human research approved the study protocol.

**Table 1:** Preoperative Characteristics of Patients with LV Aneurysm

**Variable**^**a**^	**All patients**	**Liner repair**	**Endoventricular circular patch plasty repair**
	**(n = 62)**	**(n = 26)**	**(n = 35)**
Male	38(61.3 %)	16(61.5%)	22(62.9%)
Female	24 (38.7%)	10(38.5%)	13(37.1%)
Age (years)	61.2 ± 6.8	61.8 ± 6.7	60.5 ± 6.6
Diabetes mellitus	19(30.6%)	8(30.8%)	11(31.4%)
Hypertension	40(64.5%)	16(61.5%)	24(68.6%)
LV ejection fraction	34% ± 6%	35% ± 7%	33% ± 6%
LV ejection fraction<25%	5(8.1%)	2(7.7%)	3(8.6%)
Mean NYHA class	2.6 ± 1.2	2.6 ± 1.3	2.6 ± 1.2
1-vessel disease	2(3.2%)	1(3.8%)	0(0%)
2-vessel disease	5 (8.1%)	2(7.7%)	3(8.6%)
3-vessel disease	55 (88.7%)	23(88.5%)	32(91.4%)
left main disease	14(22.6%)	6(23.1%)	8(22.9%)
Aneurysm mural thrombus	26 (41.9%)	11(42.3%)	15(42.9%)
Site of MI			
Anterior	46(74.2%)	23(88.5%)	22(62.9%)
Anteroseptal	16 (25.8%)	3(11.5%)	13(37.1%)

*P*≥0.05 except Site of MI.

^a^ For continuous variables, mean ± SD (standard deviation); for categorical variables, number (percent).

*LV* left ventricular, *NYHA* new york heart association, *MI* myocardial infarction.

### Definition of LV aneurysm

Cineangiography and echocardiography were performed preoperatively in all patients. The diagnosis of LV aneurysms was made preoperatively by the angiographic appearance (paradoxical motion), and confirmed intraoperatively. Angiographic diagnosis of LV aneurysm was based on the CASS definition (segment of the left ventricular wall protruding from the expected outline of the ventricular chamber and displaying either akinesis or dyskinesis).

### Research methods

All selected patients were divided either into group liner or patch according to the choice of the repair technique depended on factors such as localization, size and extension of the scar. The patients also were divided either into group beating heart or cardioplegia. The selected cases in Group liner included anterolateral and anteroapical aneurysms and the dimension of the scar less than 40%. The selected cases in Group patch included large anteroseptal or posterobasal aneurysms, especially the dimension of the scar more than 50%, and the cases of more severe LV damage where implantation of a patch avoids inadequate LV dimensions after the operation, facilitating better rearrangement of the myocardial fibers.

The pre-, intra- and postoperative relevant data of all selected patients were analyzed. The variables selected for analysis were the following: age, sex, diabetes mellitus, history of hypertension, functional status (NYHA, New York Heart Association), number of vessels diseased, left main disease, left ventricular function (in terms of end-diastolic and end-systolic dimensions and ejection fraction), thrombus formation in the aneurysmal cavity, site of MI (Myocardial infarction), type of repair (linear or patch),beating heart or cardioplegia, cardiopulmonary bypass (CPB) time, number of grafts, use of left internal thoracic artery (ITA),perioperative mortality, use of mechanical assist and survival. Cardiac troponin I was assayed preoperatively, and then 4 hours, 8 hours, 24 hours, 48 hours, and 120 hours postoperatively.

### Operative technique

Surgery was conducted under cardioplegia or on-pump beating heart for the aneurysm repair and for the revascularization. Under cardiopulmonary bypass, the diagnosis of dyskinetic LV aneurysms was confirmed visually and by palpation of the thinned wall of the left ventricle. If the procedure under on-pump beating heart, dispense with aortic clamping and cardioplegia, but keep the head down position and sufficient perfusion pressure for prevention of air embolism. Aortic insufficiency may necessitate the use of cardioplegic arrest during this part of the procedure. A linear LV incision was then made parallel to the left anterior descending coronary artery, 2–3 cm lateral to it, and, if present, clots were removed. Depending on the size and shape of the left ventricular cavity, a portion of the thinned wall was resected. For the linear repair, depending on the consistency of the LV wall, the edges were either sutured directly (overlaping technique) or with two strips of teflon using a combination of continuous and mattress sutures. For patch repair, the aneurysm was opened and an elliptical or circular patch of teflon, covered on the ventricular side by autologous pericardium, was sutured to the ‘red/white’ border zone inside the LV cavity, starting from the base towards the apex, using a continuous 4–0 polypropylene suture. The excess aneurysm wall was resected, leaving a residual portion that was closed using a linear repair in 2 layers, buttressed with a Teflon strip. Once ventricular repair was completed, bypass grafting was carried out. If the MR (mitral regurgitation) achieved medium, mitral valve replacement were performed to correct ischemic MR.

### Follow-up

All patients who left hospital were followed up by examination and/or their general practitioners by telephone call or a written questionnaire. Data obtained included survival, functional status and echocardiography, and cardiac-related hospital readmission. Follow-up ranged from 0 to 24 months, 2 patients (3.2%) were lost to follow-up because of migration or unknown reasons.

### Statistical analysis

Continuous variables are expressed as mean ± standard deviation. Categorical variables are presented as percentages. The Student t test was used to compare preoperative and postoperative continuous variables, the chi-squared test and Fischer’s exact test were used for analysis of categorical variables. Univariate regression analysis was used to determine factors associated with early hospital mortality and a low cardiac output state. Survival curves were calculated according to the method of Kaplan - Meier and subgroups were compared using the Log-rank test. Repeated-measure analysis of variance was used to evaluate differences over time within groups for cTnI. SPSS software version 13.0 for Windows was used in data analysis. A significant difference was considered at *p* <0.05.

## Results

### Baseline characteristics

Preoperative clinical and angiographic data are detailed in Table 1. There were 38 (61.3%) men and 24 (38.7%) women, with a mean age of 61.2 ± 6.8 years. Nineteen (30.6%) patients were diabetic, and 40 (64.5%) had hypertension. The mean NYHA class was 2.6 ± 1.2. Three-vessel disease was present in 55 patients (88.7%), 5 patients (8.1%) had double-vessel coronary disease, 2 patients (3.2%) had single -vessel coronary disease, and 14 (22.6%) had left main disease. The mean left ventricular ejection fraction (LVEF) was 34% ± 6%, 5 patients(8.1%)<25%. The major location of the aneurysm was anterior due to anterior myocardial infarction in 46 patients (74.2%).There were no significant differences between groups (linear and patch) in preoperative characteristics included age, sex, diabetes mellitus, history of hypertension, NYHA class, number of vessels diseased, left main disease, left ventricular ejection fraction, thrombus formation in the aneurysmal cavity, excepted site of MI (Anterior:linear 88.5% versus patch 62.9%, *p* <0.05; Anteroseptal:linear 11.5% versus patch 37.1%, *p* <0.05; respectively).

### Operative data

Operative data are detailed in Table 2. There were no significant differences between groups in beating heart or cardioplegia, number of grafts, use of left ITA, number of arterial grafts, CABG + MVR (Mitral valve replacement). The use of intra aortic balloon pump (IABP) and CPB time in patch group were significantly more than those in linear group (34.3% versus 23.1%, *p* <0.05; 108.3 ± 28.7 minutes versus 89.5 ± 25.6 minutes, *p* <0.05; respectively).

**Table 2:** Operative Data of Patients with LV Aneurysm

**Variable**^**a**^	**Liner repair**	**Endoventricular circular patch plasty repair**
	**(n = 26)**	**(n = 35)**
Mean no. of grafts	2.9 ± 0.7	2.7 ± 0.6
Mean no.of arterial grafts	1.4 ± 0.7	1.3 ± 0.6
Beating heart	16(61.5%)	20(57.1%)
Mean CPB time (min)	89.5 ± 25.6	108.3 ± 28.7
CPB time ≥3h	3(11.5%)	4(11.4%)
Left ITA	25(96.2%)	34(97.1%)
CABG + MVR	2(7.7%)	3(8.6%)
Isolated CABG	24(92.3%)	32(91.4%)
IABP	6(23.1%)	12(34.3%)

*P*≥0.05 except Mean CPB time and IABP.

^a^ For continuous variables, mean ± SD (standard deviation); for categorical variables, number (percent).

*CABG* coronary artery bypass grafting, *MVR* mitral valve replacement, *CPB* cardiopulmonary bypass, *ITA* internal thoracic artery, *IABP* intra aortic balloon pump.

### Clinical outcomes

Hospital mortality after SVR was 6.5% (4/62), 2 in linear group and 2 in patch group, 1 of them on the beating heart and 3 of them used of cardioplegic arrest. This included 2 patients due to low cardiac output, the other 2 patients died from multiorgan failure. An intra aortic balloon pump was required in 18 patients, of whom 16 were discharged and 2 died. Five patients underwent concomitant mitral valve replacement. Renal dysfunction occurred in 4 patients, which was managed conservatively. There were 2 patients complicated postoperative arrhythmias, 1 with cerebral complications and the incidence of postoperative infection was low (7.7%). Postoperative angina pectoris of all patients disappeared or released, 42 of them disappeared completely. The cardiac function were improved at different level, the cardiac function can recover to I~II with clinical evaluation. Factors analyzed as predictors of early mortality and postoperative low cardiac output are listed in Table 3.

**Table 3:** Univariate Analysis of Prognostic Factors for Early Mortality and Low Cardiac Output

**Variable**	**Mortality**	**Low Output**
Sex	≥0.05	0.04
Diabetes mellitus	≥0.05	0.04
Congestive heart failure	0.03	0.02
Ejection fraction≤ 25%	0.01	0.016
Absence of angina	0.032	≥0.05
LVED (mm)≥ 65	0.03	0.04
Cardiopulmonary bypass ≥3 h	0.04	0.036
Coronary artery bypass	≥0.05	0.04
Mitral valve replacement	≥0.05	0.03
IABP	≥0.05	0.034

*LVED* left ventricular end-diastolic dimension, *IABP* intra aortic balloon pump.

### Linear repair versus endoventricular circular patch plasty repair

**Figure 1:**
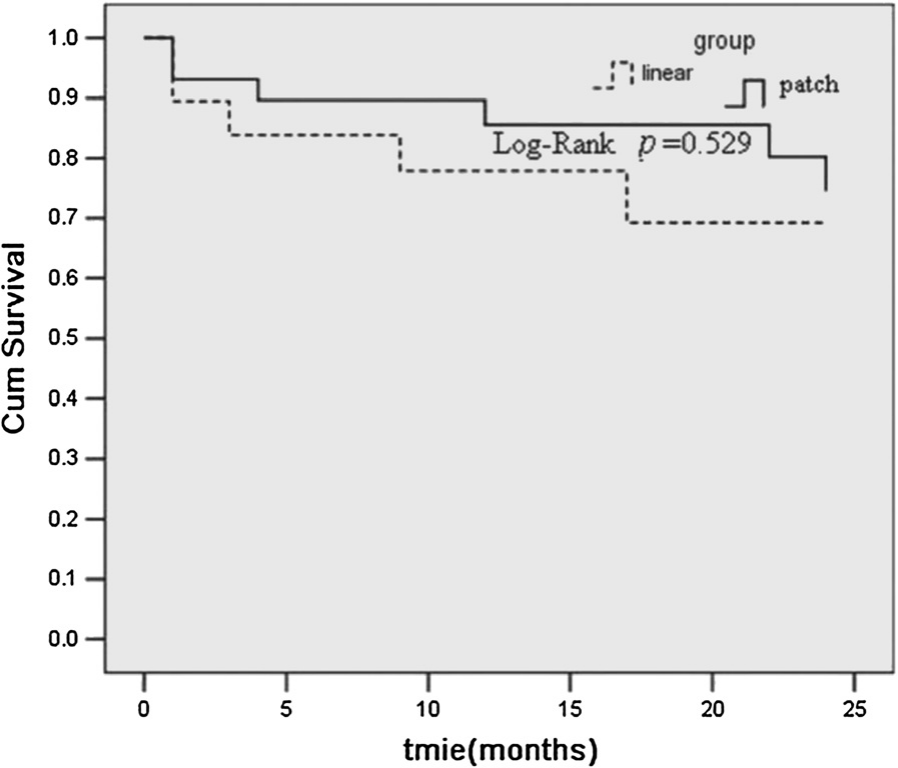
**Kaplan–Meier survival curve of patients in both groups undergoing repair of left ventricular aneurysm.***Linear* linear repair group, *Patch* endoventricular circular patch plasty repair group.

The perioperative mortality was 6.5%, and major mortality was not significantly different between linear repair and patch groups. Actuarial survival rates at 6, 12 and 24 months were 87.4, 82.5 and 72.4%, respectively. There was no significant difference in survival between the two groups (*p*= 0.529, Figure 1). Midterm postoperative echocardiographic findings showed significant improvements in left ventricular function in both groups, in terms of end-diastolic and end-systolic dimensions and ejection fraction (Table 4).

**Table 4:** Comparison of Preoperative and Midterm Postoperative Echocardiographic Parameters

**Variable**	**Liner repair**	**Endoventricular circular patch plasty repair**	***p*Value**
	**(*n*= 26)**	**(*n*= 35)**	
Ejection fraction			
Preoperative	40% ± 11%	32% ± 12%	<0.001
Postoperative	46% ± 12%	38% ± 14%	<0.001
LVED (mm)			
Preoperative	62 ± 12	69 ± 11	<0.05
Postoperative	58± 10	67 ± 11	<0.05
LVES (mm)			
Preoperative	50 ± 12	52 ± 10	<0.05
Postoperative	46 ± 7	48 ± 9	<0.05

*LVED* left ventricular end-diastolic dimension, *LVES* left ventricular end-systolic dimension.

### Beating heart versus cardioplegia

**Figure 2:**
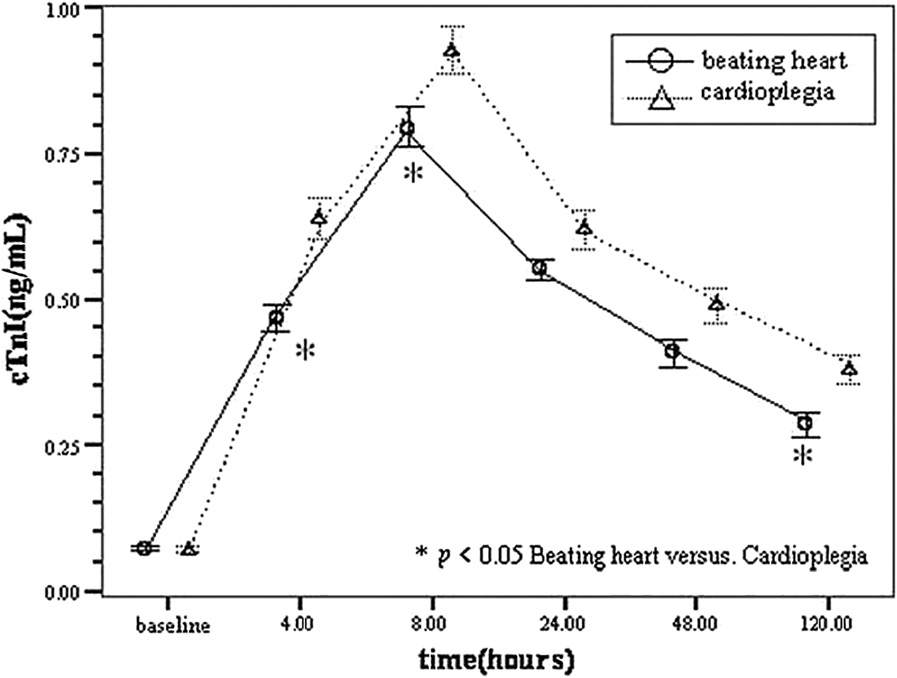
**Changes in levels of plasma cTnI over time.***Beating heart* beating heart group, *Cardioplegia* cardioplegia group, *cTnI* cardiac troponin I.

**Figure 3:**
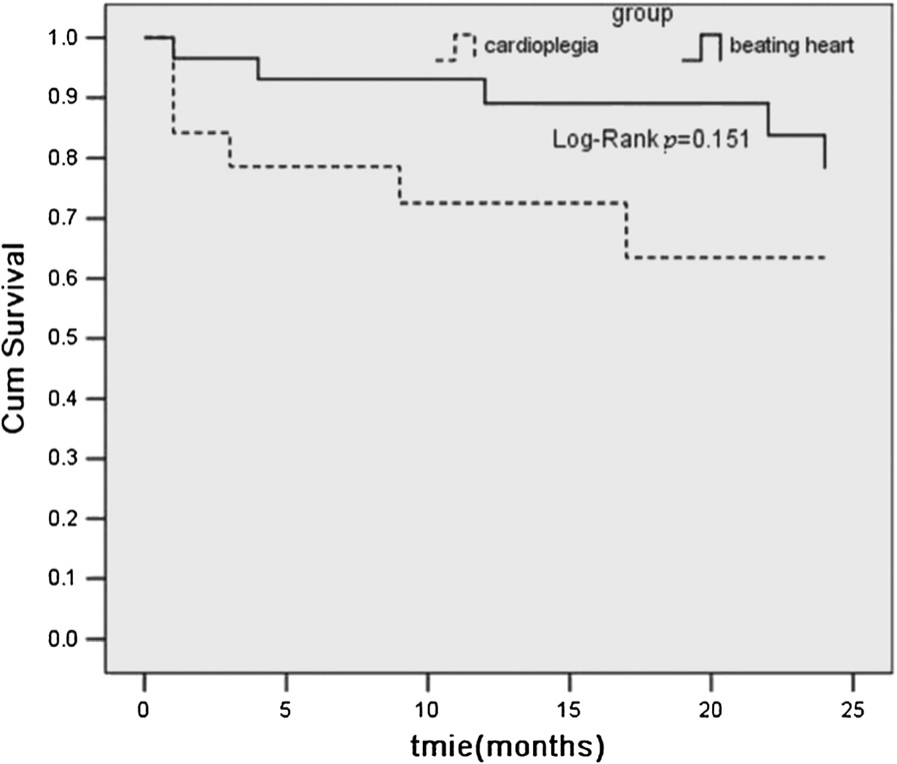
**Kaplan–Meier survival curve of patients in the beating heart and cardioplegia groups undergoing repair of left ventricular aneurysm.***Beating heart* beating heart group, *Cardioplegia* cardioplegia group.

Cardiac troponin I was assayed preoperatively, and then 4 hours, 8 hours, 24 hours, 48 hours, and 120 hours postoperatively. The pre- and postoperative relevant data of all selected patients were analyzed. As shown in the Figure 2, the cTnI increased sharply after surgery, reaching it’s peak within 8 hours, then slowly decline in both groups (beating heart versus cardioplegia). Baseline cTnI levels (preoperative cTnI levels) were similar in both groups (0.07 ± 0.009 ng/mL versus 0.068 ± 0.015 ng/mL, p= 0.817). Significant difference between the two groups were found at the postoperative time points of 4 hours, 8 hours and 120 hours (0.465 ± 0.063 ng/mL versus 0.637 ± 0.096 ng/mL, *p*= 0.004; 0.793 ± 0.09 ng/mL versus 0.927 ± 0.109 ng/mL, *p*= 0.043; 0.285 ± 0.055 ng/mL versus 0.38 ± 0.067 ng/mL, *p*= 0.023; respectively). The beating heart technique reduced postoperative peak release by 27% for cTnI compared with the cardioplegia group. The beating heart technique increased the perioperative survival by 9% compared with the cardioplegia group (97.2% versus 88.5%, *p* <0.05). Compared with the actuarial survival rates from 2 to 24 months, there was no significant difference between the groups (*p*= 0.151, Figure 3).

## Discussion

Left ventricular aneurysms are a common complication of myocardial infarction. Following a transmural MI, ventricular aneurysms develop in 10%–35% of patients [5,6]. The scarred area becomes increasingly thin and dyskinetic. The aneurysm absorbs part of the LV ejection, eventually leading to cardiac failure which may be refractory to medical therapy and require surgical treatment. When pulmonary congestive symptoms, angina pectoris, low cardiac output, malignant arrhythmias, or embolization occur, aneurysmectomy should be performed.

Surgical repair of LV aneurysms was first performed by Charles Bailey [7].The first resection under cardiopulmonary bypass was reported by Denton Cooley and associates [8]. After that, the traditional linear repair and newer patch techniques were devised, modified and reported to improve results. But controversy still exists regarding the optimal surgical technique for postinfarction left ventricular aneurysm repair. Dor indicated there was distortion of LV geometry by the linear suture, and incomplete septal infarct exclusion. In addition, the left anterior descending artery (LAD) territory could not be revascularized effectively. The geometric repair technique involved more infarct exclusion with a circular patch [4]. Vural and colleagues reported the results of patch repair were superior to those of linear repair in terms of LV geometry and long-term clinical outcomes [9]. But some retrospective clinical studies failed to demonstrate any difference between linear and patch repairs [10]. Coskun and colleagues found no significant difference in mortality between the two techniques. Patients undergoing the Dor procedure had a tendency towards higher hospital mortality but better 10-year survival. There were no differences between groups in terms of clinical status as indicated by echocardiographic data [11].

In this study, the major mortality was not significantly different between linear and patch repair groups, also the actuarial survival rates within 24 months (*p*= 0.529). Midterm postoperative echocardiographic findings showed significant improvements in left ventricular function in both groups, in terms of end-diastolic and end-systolic dimensions and ejection fraction. In this study, the choice of the repair technique depended on factors such as localization, size and dimension of the scar. The preoperative cardiac status of the two groups was not entirely identical, especially the site of MI. Hence, and that’s why we found the use of IABP and CPB time in the patch group were significantly more than those in linear group (34.3% versus 23.1%, *p* <0.05; 108.3 ± 28.7 minutes versus 89.5 ± 25.6 minutes, *p* <0.05; respectively).

Linear plasty allows wide excision of the scar area and linear closure of the LV opening within the scar, thus leaving some scar tissue. For this technique, the interventricular septum should be intact. It is advantageous in anterolateral and anteroapical aneurysms [11]. In the Dor procedure, a patch is implanted inside the LV, thereby excluding the akinetic portion of the LV septum and permitting reconstruction and restoration of LV geometry [12]. Dor plasty is effective for large anteroseptal or posterobasal aneurysms, and can be used in cases of more severe LV damage where implantation of a patch avoids inadequate LV dimensions after the operation, facilitating better rearrangement of the myocardial fibers [13]. We agreed with Pedro that the technique of repair of LV aneurysms should be adapted in each patient to the cavity size and shape, and the dimension of the scar [5]. In this study, both patch and linear repair achieved good results with respect to perioperative mortality, late functional status and survival.

Dor indicated that aneurysmectomy is contra-indicated in patients with LV ejection fraction <30% [14]. In this study, we also found that mitral valve replacement was related to low output and LVEF<25% related to low output and mortality. But we still think the LVEF<30% is not the absolute operative contraindication. As long as if only the active myocardium exist, the condition of the coronary artery is able to revascularization, CABG can improve the myocardial ischemia, paradoxical motion eliminated after SVR, the heart function can be improved. The prognosis of ischemic cardiomyopathy is more closely related to LV volume than LVEF [15]. In our study, the LVEF of 5 patients (8.1%)<25%, 3 of them obtained satisfactory therapeutic effects. In accordance with Mickleborough [16], we recommend an aggressive approach to revascularization and ventricular reconstruction in patients with coronary artery disease and poor ventricular function. Suspect the results of STICH trial, Adhyapak [17] is also convinced that endoventricular circular patch plasty improves prognosis for end-stage heart failure due to ischemic cardiomyopathy in patients not suitable for cardiac transplantation. In our study, IABP was used in 18 patients and obtained satisfactory clinical results. IABP was an important and effective therapeutic method for low cardiac output.

In this study, the beating heart technique reduced postoperative peak release by 27% for cTnI compared with the cardioplegia group (0.46 ± 0.06 ng/mL versus 0.63 ± 0.09 ng/mL, *p*= 0.004), and increased the perioperative survival by 9% (97.2% versus 88.5%), but the actuarial survival rates were not significantly different between the groups from 2 to 24 months (*p*= 0.151). Two types of myocardial injury are recognized during open heart procedures, ischemic injury and reperfusion injury. In this study, the result might imply that myocardium injury in the beating heart group would be much less than in the cardioplegia group, which indicated that beating heart technique was able in part to relieve myocardial injury in patients with LVA undergoing SVR and increased the perioperative survival,especially refer to high-risk patients with severe left ventricular dysfunction.

We believe that the advantage of beating heart technique including: ①Performing this technique under on-pump beating heart enables the surgeon to identify the contractile and non-contractile borders of the ventricular wall [18]. ② which can relieve myocardial ischemia and myocardial injury, myocardium can obtain better protection. ③ ventricular thrombus can be eliminated completely before the drainage tube was inserted into left heart. ④On open beating heart, the gas can be discharged from the incision of left ventricle, which can decrease the incidence of embolism. ⑤The LVA can be resected precisely, decrease the incidence of ventricular arrhythmia and mortality. ⑥Surgeon can confirm the tension of myocardium and the geometric shape of ventricle, which can make the repaired ventricle more similar to the normal condition and coincidence with the hemodynamic in order to improve the LV function.

LVA are often associated with total occlusion of LAD and poor collateral supply, 75% of patients have multivessel disease [12]. LVA repair should be carried out together with revascularization, even large area of LVA was resected, distal part of LAD was occluded, although small area of ventricular septum revascularized, which is important for the therapeutic results in proximal and distal stage [19,20]. LAD revascularization increases flow through the perianeurysmatic portions of the septum and lateral wall, so contributing to improved LV function [21].

It is important to note that this study had some limitations. This study included just 62 patients in a single center. The preoperative cardiac status of the two groups was not entirely identical and followed just 2 years. A final determination would require a multicentre study involving a larger sample capacity and long time follow.

## Conclusions

In conclusion, both techniques (linear and patch) achieved good results with respect to perioperative mortality, functional status and survival. The choice of surgical technique should be adapted in each patient to the cavity size and shape, and the dimension of the scar, especially the presence of an anteroseptal scarred area. The beating heart technique may to some extent relieve myocardial injury in patients undergoing SVR,especially refer to high-risk patients with severe left ventricular dysfunction.

## Abbreviations

LVA: Left ventricular aneurysm; SVR: Surgical ventricular restoration; CABG: Coronary artery bypass grafting; cTnI: Cardiac troponin I; LV: Left ventricle; NYHA: New York Heart Association; MI: Myocardial infarction; CPB: Cardiopulmonary bypass; ITA: Internal thoracic artery; LVEF: Left ventricular ejection fraction; MVR: Mitral valve replacement; IABP: Intra aortic balloon pump; LAD: Left anterior descending artery; LVED: Left ventricular end-diastolic dimension; LVES: Left ventricular end-systolic dimension.

## Competing interests

The authors declare that they have no competing interests.

## Authors’ contributions

XSW and YQM designed the study, analyzed and interpreted the data and wrote the manuscript; QJ and JZC performed the statistical analysis, analyzed the data; XZH and JF contributed to the design the study and acquisition of data; YFS and SLX helped to draft the final manuscript and added important comments to the paper. All authors read and approved the final manuscript.

## References

[B1] Vicol C Rupp G Fischer S Summer C Dietrich Bolte H Struck E Linear repair versus ventricular reconstruction for treatment of left ventricular aneurysm: a 10-year experience J Cardiovasc Surg (Torino) 1998 39 461 467 9788792

[B2] Starling RC McCarthy PM Yamani MH Mann DL Surgical treatment of chronic congestive heart failure Heart failure: a companion to Braunwald’s Heart disease 2004 Saunders, Philadelphia 717 736

[B3] LL Ml Maruyama H Liu P Mohamed S Results of left ventricular aneurysmectomy with a tailored scar excision and primary closure technique J Thorac Cardiovasc Surg 1994 107 690 698 8127098

[B4] Dor V Surgical remodeling of left ventricle Surg Clin N Am 2004 84 27 43 10.1016/j.suc.2003.12.001 15053181

[B5] Antunes PE Silva R Ferrão de Oliveira J Antunes MJ Left ventricular aneurysms: early and long-term results of two types of repair Eur J Cardiothorac Surg 2005 27 210 215 10.1016/j.ejcts.2004.11.010 15691672

[B6] Parachuri VR Adhyapak SM Kumar P Setty R Rathod R Shetty DP Ventricular restoration by linear endoventricular patchplasty and linear repair Asian Cardiovasc Thorac Ann 2008 16 401 406 18812350 10.1177/021849230801600512

[B7] Likoff W Bailey CP Ventriculoplasty: excision of myocardial aneurysm.Report of a successful case J Am Med Assoc 1955 158 915 920 10.1001/jama.1955.02960110021006 14381268

[B8] Cooley DA Collins HA Morris GC Jr Chapman DW Ventricular aneurysm after myocardial infarction. Surgical excision with use of temporary cardiopulmonary bypass J Am Med Assoc 1958 167 557 560 10.1001/jama.1958.02990220027008 13538738

[B9] Vural KM Sener E Ozatik MA Tademir O Bayazit K Left ventricular aneurysm repair: an assessment of surgical treatment modalities Eur J Cardiothorac Surg 1998 13 49 56 10.1016/S1010-7940(97)00287-X 9504730

[B10] Tavakoli R Bettex D Weber A Brunner H Genoni M Pretre R Jenni R Turina M Repair of postinfarction dyskinetic LV aneurysm with either linear or patch technique Eur J Cardiothorac Surg 2002 22 129 134 10.1016/S1010-7940(02)00210-5 12103386

[B11] Coskun KO Popov AF Coskun ST Hinz J Schmitto JD Korfer R Surgical Treatment of Left Ventricular Aneurysm Asian Cardiovasc Thorac Ann 2009 17 5 490 493 19917791 10.1177/0218492309348636

[B12] Di Mattia DG Di Biasi P Salati M Mangini A Fundarò P Santoli C Surgical treatment of left ventricular post-infarction aneurysm with endoventriculoplasty: late clinical and functional results Eur J Cardiothorac Surg 1999 15 413 418 10.1016/S1010-7940(99)00077-9 10371114

[B13] Jatene AD Left ventricular aneurysmectomy. Resection or reconstruction J Thorac Cardiovasc Surg 1985 89 321 331 3974267

[B14] Dor V Sabatier M Di Donato M Maioli M Toso A Montiglio F Late hemodynamic results after left ventricular patch repair associated with coronary grafting in patients with postinfarction akinetic or dyskinetic aneurysm of the left ventricle J Thorac Cardiovasc Surg 1995 110 1291 1299 10.1016/S0022-5223(95)70052-8 7475181

[B15] White HD Norris RM Brown MA Brandt PW Whitlock RM Wild CJ Left ventricular end-systolic volume as the major determinant of survival after recovery from myocardial infarction Circulation 1987 76 44 51 10.1161/01.CIR.76.1.44 3594774

[B16] Lynda L Mickleborough Naeem M Yves P Susan C Joan I Ventricular reconstruction for ischemic cardiomyopathy Ann Thorac Surg 2003 75 S6 S12 10.1016/S0003-4975(03)00464-8 12820729

[B17] Srilakshmi M Adhyapak Venkateswara Rao P Lessons from a mathematical hypothesis — modification of the endoventricular circular patch plasty Eur J Cardiothorac Surg 2011 39 945 951 10.1016/j.ejcts.2010.09.023 20971019

[B18] Gürsel Levent O Monica G Fabiano P Jan G On-pump beating heart Endoventriculoplasty using Autologous Endocardium for left ventricular Aneurysm repair Balkan Med J 2011 28 328 330

[B19] LL Ml Carson S Ivanov J Repair of dyskinetic or akinetic left ventricular aneurysm: results obtained with a modified linear closure J Thorac Cardiovasc Surg 2001 121 675 682 10.1067/mtc.2001.112633 11279407

[B20] Pasini S Gagliardotto P Punta G Del Ponte S Serra M Parisi F Ottino G Di Summa M Early and late results after surgical therapy of postinfarction left ventricular aneurysm J Cardiovasc Surg (Torino) 1998 39 209 215 9639006

[B21] Vauthey JN Berry DW Snyder DW Gilmore JC Sundgaard-Riise K Mills NL Ochsner JL Left ventricular aneurysm repair with myocardial revascularization: an analysis of 246 consecutive patients over 15 years Ann Thorac Surg 1988 46 29 35 10.1016/S0003-4975(10)65847-X 3382282

